# Object Manipulation with an Anthropomorphic Robotic Hand via Deep Reinforcement Learning with a Synergy Space of Natural Hand Poses

**DOI:** 10.3390/s21165301

**Published:** 2021-08-05

**Authors:** Patricio Rivera, Edwin Valarezo Añazco, Tae-Seong Kim

**Affiliations:** Department of Electronics and Information Convergence Engineering, Kyung Hee University, Yongin 17104, Korea; patoalejor@khu.ac.kr (P.R.); edgivala@khu.ac.kr (E.V.A.)

**Keywords:** anthropomorphic robotic hand, deep reinforcement learning, synergy space, natural hand poses, object grasping, object relocation

## Abstract

Anthropomorphic robotic hands are designed to attain dexterous movements and flexibility much like human hands. Achieving human-like object manipulation remains a challenge especially due to the control complexity of the anthropomorphic robotic hand with a high degree of freedom. In this work, we propose a deep reinforcement learning (DRL) to train a policy using a synergy space for generating natural grasping and relocation of variously shaped objects using an anthropomorphic robotic hand. A synergy space is created using a continuous normalizing flow network with point clouds of haptic areas, representing natural hand poses obtained from human grasping demonstrations. The DRL policy accesses the synergistic representation and derives natural hand poses through a deep regressor for object grasping and relocation tasks. Our proposed synergy-based DRL achieves an average success rate of 88.38% for the object manipulation tasks, while the standard DRL without synergy space only achieves 50.66%. Qualitative results show the proposed synergy-based DRL policy produces human-like finger placements over the surface of each object including apple, banana, flashlight, camera, lightbulb, and hammer.

## 1. Introduction

Achieving dexterous object manipulation with an anthropomorphic robotic hand much like a human hand is still a challenge in robotics. There is a growing interest in adopting anthropomorphic robotic hands for various applications in logistics and production lines [1], and to improve emerging fields of service robotics [2]. For instance, anthropomorphic robotic hands are available such as Shadow Hand [3], ADROIT [4], and RBO Hand2 [5], which allow highly dexterous movements. Controlling these anthropomorphic robotic hands is challenging due to their high degree-of-freedom (DoF) in movements [6] compared to under-actuated manipulators such as parallel grippers and vacuum grippers [7,8]. For an anthropomorphic robotic hand, with five independent fingers and their higher DoFs, the complexity of control increases accordingly. Especially for human-like grasping of an object, which requires placing the robotic fingers around the object while considering constraints to achieve a stable grasp much like human fingers. In addition, relocation using robotic hands strongly requires a stable grasping to relocate an object to a target position without dropping. In this work, we propose a method for natural grasping and object manipulation based on a novel synergy space.

Recent works have proposed deep reinforcement learning (DRL) for object manipulation. A DRL agent (i.e., policy) learns optimal actions in an environment to maximize a cumulative reward via trial-and-error [9,10]. For instance, a DRL policy was trained using a non-parametric Relative Entropy Policy to roll an object in a real-world environment using only images from a camera sensor and a three-fingered robotic hand [9]. More recently, a DRL policy using Proximal Policy Optimization was trained for achieving complex in-hand manipulation for solving a Rubik cube using a five-fingered anthropomorphic robotic hand with RGB sensor data [6]. DRL approaches without a model of the environment are challenging as they need to interact and collect large amounts of interactions (i.e., actions and states) for learning tasks. Besides, the tasks learned by DRL are difficult to generalize to different manipulation tasks (e.g., pushing and grasping). As a result, different approaches train a DRL policy with human demonstrations to help the task with prior knowledge (i.e., behavior cloning) [11,12]. In this work, a DRL policy learned object manipulation with a latent representation of natural hand poses derived from human grasping information.

Some approaches use human demonstrations to train hand manipulation skills with supervised learning methods, thereby optimizing the DRL [13,14]. For instance, in [14] human demonstrations were used to pre-train a Q-function approximator that guided the policy exploration by minimizing the error between the demonstrations and policy experience with a prioritized replay mechanism. In [15], an algorithm for training the DRL policy using an augmented cost function with demonstrations was proposed using a natural policy-gradient search algorithm. DRL policies use expert knowledge such as grasping locations with gripper poses over the object or predefined hand poses from a grasp taxonomy for a successful grasp. This approach is proven successful for grasping trained objects [16,17]. There are not many works that use high dimensional data such as 3D point clouds as priors to compute the grasp movements for object manipulation using robotic. Ji et al. [18] took an object partial point cloud and obtained a bounding box to identify the shape, pose, and orientation. Then, based on this box a policy chooses between two grasp strategies (i.e., top-down or side-grasp) and plans the final pose for the robotic hand to grasp the object that switched to position-based force control to grasp the object. Osa et al. [19] proposed a hierarchical DRL utilizing features extracted from potential areas for object grasping in a given point cloud to teach the policies proper grasp planning. The lower-level policies learned different poses to grasp an object whereas the upper-level policy learned to select a specific grasp for each object. Learning to grasp using grasping priors allows the policies to propose novel hand poses for various objects, which otherwise would be difficult to model. However, simultaneous learning of various policies in the hierarchical DRL is not a trivial task and still requires high-quality human demonstrations. As the complexity of control increases with the number of DoF from anthropomorphic robotic hands, it is hard to identify and control each DoF simultaneously. Robust control is needed that can exploit pose information from human demonstrations and grasp priors in DRL. In this context, a prior-based latent space using 3D point clouds of haptic information could improve the DRL policy to control robotic hands.

For anthropomorphic robotic hands with a high DoF, it is crucial to derive a feasible space of optimal grasping poses to control the finger movements (e.g., finger joint angles). Earlier works employ a dimension reduction of this space using Principal Component Analysis (PCA) to simplify the planning and control of a fully-actuated robotic hand by finding the correlation between fingers and joints movements. For instance, in Santello et al. [20], patterns of covariation for 15 joint sensors in a total of 57 hand postures were investigated using PCA. The principal components computed from the eigenvalues and eigenvectors of the matrix of covariance coefficients showed that using the first three principal components could account for more than 80% of the grasp variance. In addition, including some low-order components of PCA obtained from features computed from images of 3D object models improved the control for grasping objects [21,22]. Synergies represent the variance of various hand poses for grasping and the correlation between the fingers’ joint movements in a low-dimensional space. These approaches using PCA for computing the synergies restricted the hand motion to a limited number of static hand postures defined at the end of the task. This implies that improving DRL policies to find the optimal grasp configuration requires better ways to approximate the relationship between the proper hand poses and synergy space [22]. Synergies using lower-order principal components represent coarse hand opening and closing and adding a few high-order components provides a finer control [23,24,25]. To ensure a fast convergence of the RL algorithm a dimensionality reduction in the search space is needed. For such purposes, an efficient synergy space can be used as a tool in planning and controlling a fully actuated and high-DOF anthropomorphic robotic hand for complex hand manipulations.

Our proposed DRL policy generates natural hand poses for object manipulation based on a novel low-dimensional synergy space. First, we obtained high-dimensional point clouds using haptic contact areas of various objects from human demonstrations of successful grasping. Then, we generated a novel low-dimensional synergy space from this haptic information via Continuous Normalizing Flow (CNF) using a deep autoencoder network. The obtained latent space via CNF contains a rich representation of haptic information, which a deep neural network regressor utilizes to estimate the joint angles for an anthropomorphic hand. The proposed synergy-based DRL performs a dexterous grasping and relocation of objects using an anthropomorphic robotic hand. Through various experiments, our results demonstrate that the proposed DRL policy and regressor can generate natural hand poses to grasping and relocation variously shaped test objects. The remainder of this paper is organized as follows. Section 2 provides an overview of the database, the proposed DRL structure, and the CNF synergy space modeling. Section 3 presents the qualitative and quantitative results with the synergy-based DRL and standard DRL for grasping and relocation tasks. Lastly, Section 4 presents conclusions and a discussion of the work.

## 2. Methods

**Figure 1:**
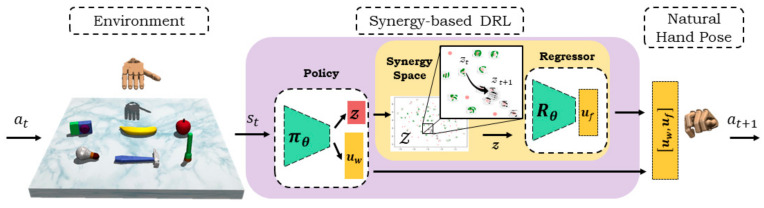
The proposed synergy–based DRL for natural object manipulation with a synergy space of object haptic information and a deep regressor to derive natural hand poses of the ADROIT anthropomorphic robotic hand.

The proposed DRL method and the simulation environment are illustrated in Figure 1. The DRL policy is instantiated using a deep neural network trained to control the robot hand movements to grasp an object and relocate to a target location. Formally, in each timestep t, the action vector $a_{t}$ controls the displacement of the joint angles in the hand and the position of the robotic hand to navigate within the environment. The robot hand interacts with the object in the environment which generates the next timestep state $s_{t}$ of the object and robotic hand (i.e., joint angles and cartesian positions), which are sent to the DRL agent $\pi_{\theta }$ (i.e., policy). The policy generates two outputs: an object-specific sample described by the synergy space of haptic information z∈Z and a vector to control the robot hand position $u_{w}$ for the next timestep t+1. The optimal natural hand pose (i.e., joint angles) $u_{f}$ is obtained via a pretrained regressor $R_{\theta }$ using the synergistic information in z. The action vector for the next step $a_{t+1}$ is composed by $\left(u_{f},u_{w}\right)$ and sent to the robot to interact within the environment again. By iterating this process, the DRL algorithm learns to derive an optimal hand pose for natural manipulation of the objects with the anthropomorphic robotic hand.

### 2.1. Simulation Environment

The simulation environment is set up with the Mujoco physics engine [26]. Mujoco is a robotics simulation package providing robotic kinematics, objects models, and manipulation tasks. The simulated anthropomorphic robotic hand is modeled after the ADROIT hand [4], which has 24-DoF with five fingers for dynamic and dexterous manipulations. The first, middle, and ring fingers have 4-DoF. The little finger and thumb have 5-DoF, while the wrist has 2-DoF. Each joint is equipped with a joint angle sensor and a haptic sensor. The movements are controlled via a position control actuator.

### 2.2. Synergy Space of Haptic Information

The proposed low-dimensional synergy space is created by encoding high-dimensional 3D haptic point clouds of each variously shaped object from successful grasping. This low-dimensional representation of haptic information helps to reduce the action space that the DRL policy explores to learn how to manipulate objects. An encoder-decoder architecture is trained with a database of grasping demonstrations to attain the latent representation. 

#### 2.2.1. PCA based Synergy Space

Previous works that derived a latent representation for grasping poses in [20,21,27,28], showed that the joints angles in the hand are not independently controlled. Also, the grasping poses can be represented in a low-dimensional synergy space. As a result, reducing the complexity of higher DoFs in anthropomorphic hands allows controlling grasp poses more effectively. However, the low-dimension representation of grasps was obtained using PCA on a set of handcrafted features in terms of hand joint angles of the predefined hand poses according to the object shape. In contrast [20,21,23], our proposed method replaces the predefined set of hand joints with higher dimensional 3D point clouds of haptic information. The haptic information includes various shapes from an apple, camera, flashlight, hammer, banana, and lightbulb extracted from a public database of human demonstrations with a large variety of successful grasps, which yields a dataset containing a rich variety of natural hand poses. Nevertheless, obtaining a linear combination of components that covers most variance in the point clouds of 3D objects shapes using PCA might be limited. PCA assumes normally distributed data and might fail to approximate the complex manifold of the natural hand poses in the database. Therefore, in this work, we utilize a deep neural network based on flow models that learns a non-linear parametric function to capture the underlying variability of such high-dimensional data. It is expected that this approach better exploits the latent representation from point clouds for generating natural hand poses.

#### 2.2.2. Contact Map Dataset

The ContactDB database contains data of various household objects including 3D point clouds of hand contact information from grasping demonstrations by human hands. A thermal camera was used to record the after-heat prints from a human hand after grasping each object [29,30]. The thermal images represent the hand-object contact map and the contact points are recorded as continuous values defined over the mesh surface for each object. The point clouds included samples from six objects, grasped with the intent of use, and handoff the object after grasping by 50 participants. We normalized the point clouds between a range of 0 and 1 and sampled a fixed number of points N=1024 from the values above a threshold of 0.75, which indicates a higher grasping probability. The 3D point clouds of haptic contact points are $X={\left\{x_{i}\right\}}_{i=1}^{N=2048}$. After sampling, each point cloud is augmented with random rotations up to 162-point clouds are obtained for each of the six objects used in this study.

#### 2.2.3. Synergy Space via Continuous Normalizing Flow

We propose a framework of condensing high-dimensional 3D point clouds into a latent space which includes local variations and complicated distribution of haptic information from human object grasping demonstrations. For such purpose, an encoder-decoder network using a flow-based model is proposed. Flow-based models are efficient to model a complex distribution (e.g., 3D point clouds of natural hand poses) into a compact space through a sequence of invertible transformation functions [31]. In particular, we can though of a point cloud from the haptic maps X to be part of a larger distribution of haptic information $Q\left(X\right)$. The continuous normalizing flow could sample points from a generic prior distribution (e.g., Gaussian) and induce a transformation to match the distribution of X [31,32]. The latent space derived from a database of natural hand poses using the encoder-decode model proposed is named a synergy space.

The encoder network $q_{\phi }\left(𝓏|X\right)$, based on the PointNet architecture [33], infers a posterior 𝓏∈Z (i.e., the sample of synergy space) given the input of haptic point clouds x∈X. The encoder is a neural network with four 1D convolutional layers with feature maps of 128, 128, 256, and 512 respectively, which are followed by three fully connected layers with 256, 64, and 4 units. The encoder architecture includes max-pooling layers as symmetric function and T-Net modules for input and feature alignment networks as introduced in the PointNet network. The decoder, $p_{\phi }\left(x|𝓏\right)$ is based on the continuous normalizing flow (CNF) architecture in [34]. It computes the reconstruction of the point cloud x conditioned on the latent space 𝓏 evaluating the likelihood of $p\left(z\right)$ and the inverse CNF, $p_{\phi }^{-1}\left(x\right)$. The decoder is instantiated with a tri-layered fully connected network with 512 units in each layer.

CNF employs a sequence of invertible transformations, defined using neural networks, that learns a mapping from the synergy space z∈ Z to the input x∈X. This mapping is done such that the embedding $z=f^{-1}\left(x\right)$ corresponds to $x=f\;\left(z\right)$. The normalizing flow f is a parametrized function that models the probability density of $p\left(x\right)$ to estimate the contact locations by minimizing:

(1) $$\left(X\right)=\frac{1}{\left|X\right|}\overset{\left|X\right|}{\underset{i=1}{\sum }}\log\left(p\left(x_{i}\right)\right)$$

For a single deterministic sample x, using a change of variables, inverse function theorem, and logarithm properties, it can be written as [32]:

(2) $$log\left(p\left(x\right)\right)=log\left(p\left(z\right)\right)+\overset{k}{\underset{i=1}{\sum }}log\left|det\left(\frac{\partial f_{i}\left(h_{i-1}\right)}{\partial h_{i-1}}\right)\right|$$

where the partial derivative is the Jacobian matrix of a function $f_{i}$ at step $h_{i-1}$ and the value of log-determinant measures the change in the log-density made by transformation $f_{i}$. In practice, choosing transformations whose Jacobian is a triangular matrix achieves a fast calculation and ensures invertibility. The density function for the synergy space 𝓏 can be defined as a spherical multivariate Gaussian distribution $p\left(z\right)=N\left(𝓏;0,\;I\right)$. In the case of continuous 3D point clouds with a continuous dense distribution $p\left(x\right)$ and a synergy space prior distribution $p\left(𝓏\right)$, CNF can be written as [34]:

(3) $$x={𝓏}_{t_{0}}+\int_{t_{0}}^{t_{1}}f\left({𝓏}_{t},t\right)dt,\;{𝓏}_{t_{0}}\sim p\left(𝓏\right)$$

(4) $$\log p\left(x\right)=\log p\left({𝓏}_{t_{0}}\right)-\int_{t_{0}}^{t_{1}}\mathrm{Tr}\left(\frac{\partial f}{\partial {𝓏}_{t}}\;\right)dt$$

Here, $z_{t_{0}}$ can be calculated using the inverse flow of the transformation function ${𝓏}_{t_{0}}=x+\int_{t_{1}}^{t_{0}}f\left({𝓏}_{t},t\right)dt$. A black-box ordinary differential equation (ODE) solver can be applied to estimate the outputs and the inputs gradients of continuous normalizing flow [35].

The synergy space was built considering the haptic contact map dataset to be a part of a larger distribution of human-like finger placements for natural hand poses. By learning a synergy space of this larger distribution via CNF, new haptic contact points are proposed similar to the contact map dataset.

#### 2.2.4. Training and Validation Synergy Space via CNF

Previous works that derived a synergy space for high DoF robotic hands selected the higher principal components from PCA to represent the variability of grasp poses [20,21,22,23,25]. Despite the dimensions differing between all works, a minimum of three or four principal components was commonly used (in some cases added more dimensions for finer control). Therefore, the CNF latent space is trained and tested for optimal dimension starting with 4 and increasing to 16, and 32.

The point clouds from the contact map dataset were randomly split into 90% training and 10% testing sets. CNF is trained using a black-box ODE solver to compute the gradients of the normalizing flows and updates the parameters of the deep network. During testing, we computed the Chamfer distance (CD) and the earth mover’s distance (EMD) between the ground truth point clouds and the reconstructed output from the CNF decoder. The lower value for these metrics indicates a better reconstruction capability of the decoder, which are defined as follows:

(5) $$\mathrm{CD}\left(X,Y\right)=\underset{x\epsilon X}{\sum }\underset{y\epsilon Y}{\min}\|x-y{\|}_{2}^{2}+\underset{y\epsilon Y}{\sum }\underset{x\epsilon X}{\min}\|x-y{\|}_{2}^{2}$$

(6) $$\mathrm{EMD}\left(X,Y\right)=\underset{\phi :X\to Y}{\min}\sum_{x\epsilon X}\|x-\phi \left(x\right){\|}_{2},$$

where $x\in X^{N}$ and $y\in Y^{N}$ are two point clouds with the same dimension and ϕ is a bijective function between them.

### 2.3. Synergy-Based Regressor

The regressor estimates the corresponding joint angles for the hand pose sampled from the low-dimensional synergy space.

#### 2.3.1. Haptic Map Dataset

A dataset of natural hand poses from human demonstrations and 3D point clouds of object contact points was created. Retargeting hand poses obtained from human demonstrations into our simulated environment, the haptic sensors located in the simulated anthropomorphic robotic hand provided the approximate locations of contact points over the object mesh. Then, a normal distribution of points was sampled from the object surface as 3D point clouds, $h^{\prime }={\left\{h_{i}\right\}}_{i=1}^{N=2048}$. Complementarily, the joint angles corresponding to the 22-DoF of the hand pose while grasping an object, $u^{\prime }={\left\{u_{i}\right\}}_{i=1}^{22}$ were recorded. The generated dataset $\left(h^{\prime },u^{\prime }\right)$ had 2730 pairs of 3D contact point clouds and joint angles for 22-DoF hand poses for each object. This dataset was named the haptic map dataset and used in training the regressor.

#### 2.3.2. Deep Regressor Using Synergy Information

**Figure 2:**
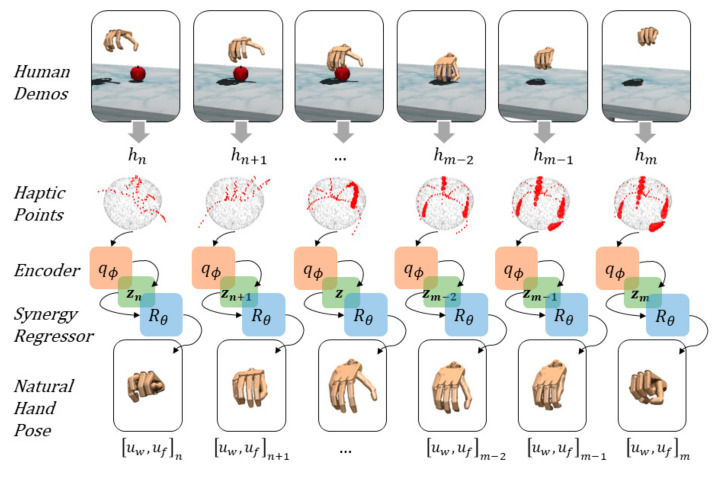
From human demonstrations (top row), haptic information is shown in point clouds (second row). CNF encodes the haptic information, $q_{\phi }$ into a synergy space representation, 𝓏. The deep regressor, $R_{\theta }\left(𝓏\right)$ estimates natural hand poses (final row) in time series.

The 3D point clouds from the haptic map dataset ${h^{\prime }}_{i}$ are mapped into synergy representations using the pre-trained encoder, ${𝓏}_{h}=q_{\phi }\left(h_{i}\right)$. Then, the regressor, $R_{\theta }$ uses these latent representations to estimate the joint angles, $u_{f}$ generating a natural hand pose of the anthropomorphic hand. The regressor is parameterized using a neural network with four layers of 256 neurons. Each layer uses a ReLU piecewise activation function and batch normalization. The parameters are optimized minimizing the mean squared error (MSE) between the regressor output and corresponding joint angles $u^{\prime }$ for the 22-DoF fingers of the anthropomorphic hand. Figure 2 illustrates the regressor under a DRL policy, $\pi_{\theta }$, using a synergy representation from the point cloud to estimate the angles of the finger joints for corresponding natural hand poses in time series. 

#### 2.3.3. Training and Validation Synergy-Based Regressor

The haptic map dataset was randomly split into 80% for training and 20% for testing. Training of the regressor minimizes the MSE loss function using Adam optimizer with a learning rate of $3\times 10^{-4}$. For testing, the overall MSE error was computed for each joint angle of the anthropomorphic hand with the regressor prediction. The regressor was trained until the error for every joint is less than 5% from each corresponding DoF.

### 2.4. Synergy-Based Deep Reinforcement Learning

In our system, the DRL policy explores potential solutions for natural object grasping and relocation using the anthropomorphic robotic hand. The policy accesses the synergy space corresponding to each object and obtains low-dimensional synergistic haptic information. Finally, the pre-trained regressor, $R_{\theta }$ estimates a natural hand pose based on the synergistic information. The goal of the policy, $\pi_{\theta }$ is to maximize the sum of expected rewards at the end of each training episode and utilize a reward function as an indicator of task completion. 

A model-free DRL policy $\pi_{\theta }\left(a_{t},s_{t}\right)$ is proposed, where the action vector $a_{t}$ is an element of ${𝓏}_{h}$ and represents the haptic maps in the synergy space. $s_{t}$ is the current state of the simulation with the Cartesian coordinates of the object, hand, and target location within the environment. A reward function, $r_{t}=R\left(s_{t},a_{t}\right)$ provides a measure of the completeness of the task, measures how close is the object to the target location at the end of an episode. For training, an on-policy gradient method optimizes the parameters, θ of the policy by maximizing the objective, $J\left(\theta \right)$ using gradient ascent. Our implementation follows the natural policy gradient implementation (NPG) in Kakade [36], which computes the vanilla policy gradient:

(7) $$\nabla_{\theta }J\left(\theta \right)=\frac{1}{NT}\overset{N}{\underset{i=1}{\sum }}\overset{T}{\underset{t=1}{\sum }}\nabla_{\theta }log\pi_{\theta }\left(a_{t}^{i}|s_{t}^{i}\right)A^{\pi }\left(s_{t}^{i},a_{t}^{i}\right)$$

Then, it pre-conditions this gradient with the Fisher Information Matrix, $F_{\theta }$ and makes the following normalized gradient ascent update:

(8) $$\theta_{k+1}=\theta_{k}+\sqrt{\frac{\delta }{\nabla_{\theta }J{\left(\theta \right)}^{T}\cdot \;F_{\theta_{k}}^{-1}\cdot \nabla_{\theta }J\left(\theta \right)}}\cdot F_{\theta_{k}}^{-1}\cdot \nabla_{\theta }J\left(\theta \right)$$

where δ is the step size of choice. The advantage function $A^{\pi }$ is the difference between the value for the given state-action pair and value function of the state, the NPG implementation in [36] uses the general advantage estimator (GAE) defined as:

(9) $$A^{GAE}=\sum_{l=0}^{T}{\left(\gamma \lambda \right)}^{l}\delta_{t+l}^{V}$$

where, $\delta_{t}^{V}=r_{t}+\gamma V\left(s_{t+1}\right)-V\left(s_{t}\right)$ defines the temporal difference residual between consecutive prediction assuming a value function V approximating the true value function, $V^{\pi }$. Both the policy and value networks share the same architecture and were defined by a three-layer neural network with 64 units in the hidden layers.

#### 2.4.1. DRL Reward for Task Completion

Shaping the reward function to solve the manipulation tasks must reflect the progress on the learning problem and guide the exploration of DRL. In our work, the reward is defined as a combination of the Cartesian distance between the anthropomorphic hand, object, and target locations. The following distances are used: $r_{po}$ is the distance from the robotic hand to the object location, $\;r_{pt}$ from the robotic hand to the relocation target, and $r_{ot}$ from the object location and relocation target. Then the total reward is given as:

(10) $$r_{goal}=-r_{po}+\left(1-r_{pt}-r_{ot}\right)\;*\;I\left(o_{z}>\lambda_{th}\right)$$

During training, the term $r_{po}$ aims to reduce the distance between the hand and the object. Then, the second term evaluates if the object $o_{z}$ has been lifted from the table above a threshold $\lambda_{th}$. When the object is lifted, minimizing the distance between the hand and object to the target location receives a higher reward. The total reward, $r_{goal}$ measures the completion of grasping and relocation tasks. The greater the sum of rewards at the end of an episode indicates the faster the policy solves the tasks.

#### 2.4.2. Training and Validation for Synergy-Based DRL

Using our proposed approach, an individual policy in a multilayer perceptron with two hidden layers of 64 nodes was trained three times per object. All policies were trained for 5000 iterations, each iteration with n=200 episodes, and a time horizon of T=1000 time steps. The parameter initialization for each policy was done using a random seed every time. The proposed method is compared against a standard DRL trained per object without the synergy space to solve the same grasping and relocation tasks using the NPG algorithm and the reward function $r_{goal}$. The training also used the same number of iterations, episodes, and time steps. We evaluate the success rate between both methods. The success rate is the ratio of successful grasping and relocation among all generated trajectories. We compare the success rate for the standard DRL using NPG without the synergy space and the proposed DRL using synergy space from 100 generated trajectories.

## 3. Results

This section describes the qualitative and quantitative results for each component of the proposed method. 

### 3.1. Validation of the Synergy Space

**Figure 3:**
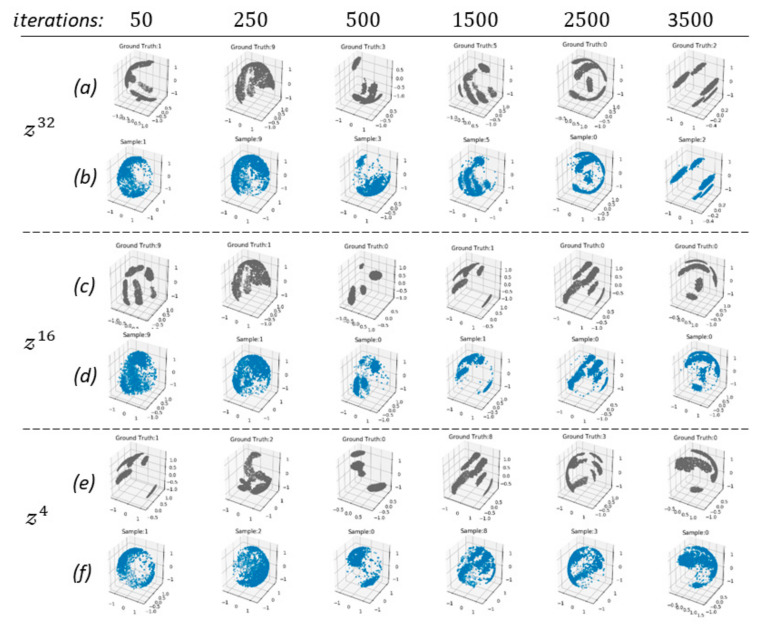
Reconstruction of object haptic information using the synergy-based encoder-decoder via CNF for three synergy dimensions. The illustrations are derived from training iterations: (**a**), (**c**), and (**e**) point clouds shown in gray are the ground truth haptic maps, the (**b**), (**d**), and (**f**) point clouds in blue are the reconstructed maps from CNF models with dimensions of $z^{32},z^{16},z^{4}$, respectively.

The qualitative results in Figure 3 show the point cloud reconstruction produced by the CNF decoder. Random test samples from the contact map dataset were used for three synergy space dimensions. For each synergy dimension, the input point clouds are illustrated in the rows of Figure 3a, and the CNF decoder reconstruction is illustrated in the rows of Figure 3b. Testing reconstruction using the decoder after the initial iterations (i=50) resulted in a random point cloud for all synergy dimensions. After (i=3500) training, the reconstruction using a synergy space ${𝓏}^{32}$ is less noisy than the other two. However, it is shown that all three synergy dimensions are capable of encoding a wide variability of haptic information encountered in the contact map dataset.

In Table 1, we show the average CD and EMD distance errors between the ground truth and reconstruction using the contact map test dataset for the initial and final training iterations. The initial CD and EMD errors provide a reference point to compare the improvement in reconstruction for each synergy dimension. It is noticed that after training, there was not a notable difference between the results obtained using ${𝓏}^{32}$, ${𝓏}^{16}$, and ${𝓏}^{4}$. Although, ${𝓏}^{32}$ produces a slightly better reconstruction in terms of CD and EMD, the difference between ${𝓏}^{32}$ and ${𝓏}^{4}$ is not very significant, respectively. Therefore, ${𝓏}^{4}$ was used in the later experiments.

### 3.2. Validation of the Synergy-Based Deep Regressor

**Figure 4:**
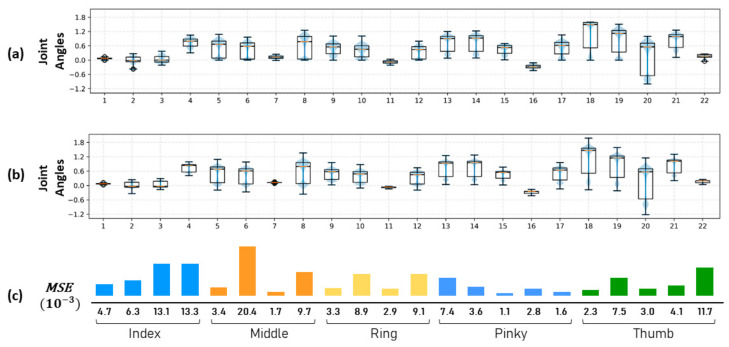
The mean squared error (**c**) between the joint angle distributions of the haptic map dataset, (**a**) and the angles distributions estimated by the regressor (**b**) for each finger in the anthropomorphic hand. A lower error indicates higher confidence in estimating the joint angles of a natural grasping pose.

To validate the deep regressor, the ground truth joint angles from the haptic map dataset were compared to the regressor output. For each finger of the anthropomorphic hand, the joint angle distribution from the haptic map dataset is illustrated in Figure 4a,b the joint angles estimated by the synergy-based regressor. The difference between both is shown in Figure 4c, where the largest error from all joints is $20.4\;\times 10^{-3}$ in the second joint of the middle finger and the proportional error is below 2% of the total DoF range. For the DoF of every finger in the anthropomorphic hand, the angular different error is kept below 5%.

### 3.3. Object Grasping and Relocation via Synergy Based DRL

The average success rates of the given tasks per object using the standard DRL without synergy and proposed synergy-based DRL are shown in Table 2. The overall synergy-based DRL success rate was 88.38%, outperforming the 50.66% achieved by the standard DRL without synergy. For the objects including lightbulb, flashlight, and camera, the standard DRL could not learn proper hand poses for solving the manipulation tasks. In contrast, the proposed synergy-based DRL policy achieved a higher success rate for all objects. The success rate from the camera was lower due to the orientations where the major *axis* of the camera aligned with the sagittal plane of the robotic hand, and the synergy-based DLR had trouble positioning the robotic hand for grasping. 

**Figure 5:**
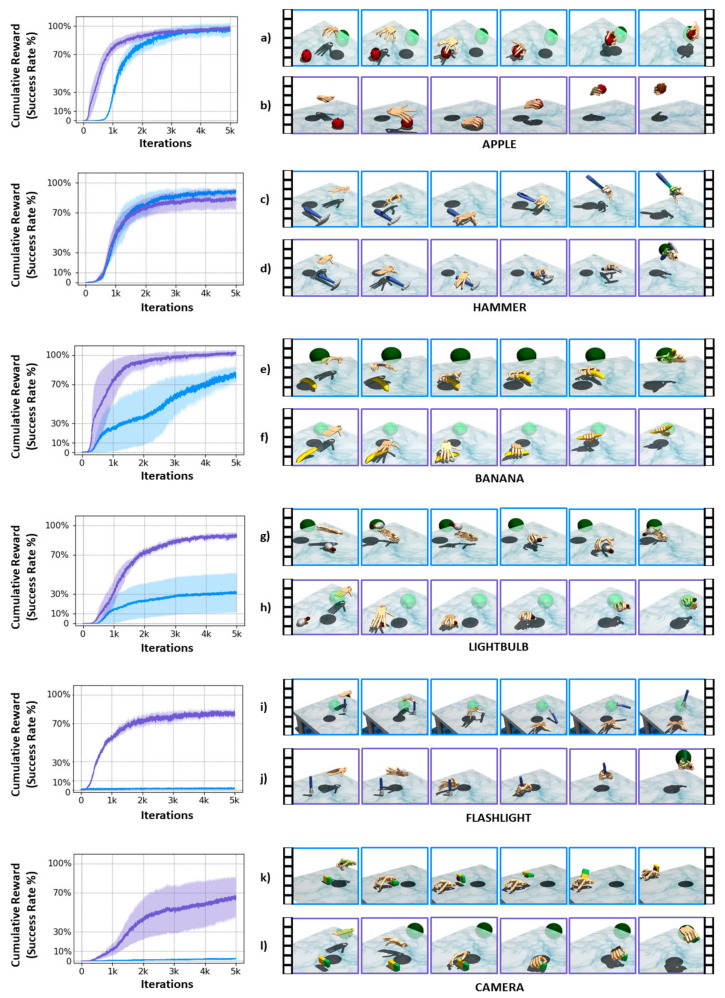
The left column shows the average sum of rewards from training the manipulation task with the proposed synergy-based DRL in purple and synergy-less standard DRL in blue. The solid line represents the mean and the shadow area of the standard deviation of the rewards. The right column shows the time-series frames from grasping and relocation of each object from apple, banana, hammer, lightbulb, flashlight, and camera with the standard DRL in the blue frames of (**a**,**c**,**e**,**g**,**i**,**k**), and the proposed synergy-based DRL in the purple frames of (**b**,**d**,**f**,**h**,**j**,**l**).

Figure 5 shows the average cumulative reward for all objects (left) and time series frames of the DRL policies during grasping and relocation (right). Overall, the cumulative reward from the synergy-based DRL grew faster and higher compared to the standard DRL. This implies that the proposed DRL learned to grasp and relocate the object with fewer iterations and maintain the object in the target location for a longer time. However, the hammer cumulative reward for the standard DRL is slightly higher than the proposed DRL. Because the standard DRL grasps the hammerhead and moves to the target location faster compared to the proposed DRL, Figure 5c,d, that accumulates a larger value for the second term of the reward function in Equation (10). 

For objects such as apple, hammer, and banana, the standard DRL generated unnatural hand poses as seen in the row frames of Figure 5a,c,e. In contrast, our system generated a pre-shape of the hand extending the fingers, seen in the row frames of Figure 5b,d,f, and then proceeded to close the hand grasping the object. In the particular case of the lightbulb in Figure 5h, the size of the object sometimes made the proposed DRL lose its grasp. The standard DRL generally manipulates with a wide range of unnatural behaviors, including hitting the object hard as shown in the row frames of Figure 5i. This is because DRL merely attempts to increase the reward only. However, this phenomenon is part of the learning process of DRL while exploring the environment. By including prior knowledge as done in this work, our proposed DRL prevents and avoids these unnatural behaviors.

## 4. Discussion

A robust DRL with anthropomorphic robotic hands requires learning an effective policy for controlling a robot hand with large DoFs. By learning from human grasping demonstrations, we can leverage the prior knowledge needed to learn various tasks with DRL for robotic manipulation. In this work, we have shown that integrating a synergy space derived from haptic information into DRL improves the learning for object manipulation. First, deriving the latent space via CNF showed promising results, reconstructing the complex distribution of high-dimensional point clouds as in Figure 3. We explored empirically the dimension of the latent space and analyze that a 4-dimensional synergy space could encode as much information as a 16- and 32-dimensional synergy space. Further analysis of the synergy dimension could provide a perspective of when is more beneficial to use a more expressive (i.e., larger) latent dimension.

The policies trained via the proposed method outperform in five out of six objects tested as in Figure 5. The hand poses estimated by the proposed regressor are correlated with the variety of natural grasps in the grasping demonstration database used for deriving the synergy space. The proposed DRL could be used for grasping objects with similar shapes to the objects in the training database. For more complex and novel objects, our method could be fine-tuned with an additional synergy space of them.

The DRL manipulation system controls the actions of a robotic hand with high dexterity for grasping and relocating various objects. The manipulation results show the hand extending the fingers before reaching the object and adapt the angle for grasping as seen in Figure 5. Furthermore, the abnormal behaviors for object manipulation observed with the standard DRL were avoided with our proposed DRL.

## 5. Conclusions

This work proposed a synergy-based DRL policy for generating natural hand poses for grasping and relocating variously shaped objects including apple, hammer, banana, lightbulb, flashlight, and camera. The average success rate achieved by the proposed DRL policy was 88.38%, outperforming the 50.66% success rate achieved by standard DRL without synergy. We trained an encoder-decoder network via CNF to reduce high-dimensional 3D point clouds of hand poses into a 4-dimensional synergy space. Then, a synergy-based regressor estimated the joint angles for the 22-DoFs from the anthropomorphic finger joints with less than a 2% MSE error. Finally, the proposed method for grasping and relocation using the anthropomorphic hand showed qualitative and quantitative results, indicating its ability to produce natural movements for manipulating variously shaped objects.

Effectively reducing the action space for DRL via synergies helped to learn a policy for natural grasping with a 22-DoFs anthropomorphic hand. Learning an individual synergy space for each object allows the proposed DRL to produce natural hand poses and grasp the objects in a human-like manner (i.e., opening the hand before grasping and placing the fingers on the object in a natural way). Finally, an extension of this work would be learning a general synergy space for various objects conditioning the latent space to the corresponding object shape. To learn a non-trivial grasping and relocation policy with natural hand poses for an anthropomorphic hand, future considerations include experiments with more complex objects and improving the synergy space representation for more complicated objects.

## Figures and Tables

**Table 1:** The mean value of CD and EMD reconstruction errors by the CNF decoder for three synergy dimensions.

Synergy Space Dimension	Iteration No. 50		Iteration No. 3500	
	CD	EMD	CD (×10^−3^)	EMD (×10^−2^)
${𝓏}^{32}$	2.915	1.588	0.219	0.896
${𝓏}^{16}$	2.923	1.590	0.269	0.844
${𝓏}^{4}$	2.908	1.585	0.465	0.989

**Table 2:** The success rate (%) of grasping and relocating tasks using the standard DRL without synergy and the proposed synergy-based DRL.

Objects	Standard DRL without Synergy (Mean ± Std)	Proposed Synergy-Based DRL (Mean ± Std)
Apple	85.33 (±6.03)	93.66 (±2.08)
Banana	80.67 (±8.33)	90.00 (±4.73)
Hammer	92.33 (±6.51)	95.33 (±4.13)
Lightbulb	21.67 (±6.31)	86.50 (±5.51)
Flashlight	9.00 (±1.73)	88.16 (±2.52)
Camera	15.00 (±1.53)	76.67 (±4.62)

## Data Availability

A publicly available dataset was analyzed in this study. This data can be found here: GitHub—samarth-robo/contactdb_utils: Python and ROS (C++) utilities for the ContactDB dataset (5 August 2021).
